# A dietary pattern derived using B-vitamins and its relationship with vascular markers over the life course

**DOI:** 10.1016/j.clnu.2018.06.969

**Published:** 2019-06

**Authors:** Jane Maddock, Gina L. Ambrosini, Julian L. Griffin, James A. West, Andrew Wong, Rebecca Hardy, Sumantra Ray

**Affiliations:** a MRC Lifelong Health & Ageing at UCL, 33 Bedford Place, London WC1 B5JU, United Kingdom; b MRC Elsie Widdowson Laboratory, Cambridge CB1 9NL, United Kingdom; c NNEdPro Global Centre for Nutrition and Health (Affiliated with: Cambridge University Health Partners, Wolfson College Cambridge and the British Dietetic Association), St John's Innovation Centre, Cowley Road, Cambridge CB4 0WS, United Kingdom; d School of Population and Global Health, The University of Western Australia, 35 Stirling Highway, Crawley 6009, Perth, Western Australia, Australia; e Department of Biochemistry, Tennis Court Road, University of Cambridge, Cambridge, CB2 1GA, United Kingdom

**Keywords:** Reduced rank regression, Dietary patterns, Homocysteine, Folate, Intima-media thickness, Pulse wave velocity

## Abstract

**Background:**

Diet may influence vascular function through elevated homocysteine (Hcy) concentrations. However the relationship between dietary patterns (DP), characterised by Hcy and its associated nutrients is unknown.

**Objective:**

To identify a DP characterised by plasma Hcy, dietary folate and dietary vitamin B12, and examine its associations with two markers of vascular function: carotid intima-media thickness (cIMT) and pulse wave velocity (PWV).

**Methods:**

1562 participants of the MRC National Survey of Health and Development (NSHD), a British birth cohort, with dietary data measured at least once between 36 and 60–64 years, and cIMT or PWV measured at 60–64 years were included. DPs were derived using reduced rank regression with three intermediate variables: 1) plasma Hcy (μmol/L) 2) folate intake (μg/1000 kcal) 3) vitamin B12 intake (μg/1000 kcal). Multiple regression models assessed associations between the derived DP z-scores and vascular function adjusting for dietary misreporting, socioeconomic position, BMI, smoking, physical activity and diabetes.

**Results:**

A DP explaining the highest amount of shared variation (4.5%) in plasma Hcy, dietary folate and dietary vitamin B12 highly correlated with folate (*r* = 0.96), moderately correlated with vitamin B12 (*r* = 0.27), and weakly correlated with Hcy (*r* = 0.10). This “high B-vitamin” DP (including folate) was characterised by high intakes of vegetables, fruit and low fibre breakfast cereal, and low intakes of processed meat, white bread, sugar and preserves. No associations were observed between DP z-scores and vascular function at any time point following adjustment for covariates.

**Conclusion:**

This study explored a specific hypothesised pathway linking diet to vascular function. Although we found no consistent evidence for an association between a high B-vitamin DP and vascular function, we did observe an association with CRP and triglycerides in secondary analyses. Further analyses using strongly correlated and biologically relevant intermediate variables are required to refine investigations into diet and CVD in longitudinal cohort data.

## 1 Introduction

Decades of research have led to the identification of risk factors for cardiovascular disease (CVD) and the development of effective treatment strategies. Despite this progress [1], CVD remains a significant public health concern worldwide [2]. Identifying environmental factors that influence the early stages of CVD will support prevention strategies. Examining vascular endothelial dysfunction, which signals atherosclerosis, can support this goal [3]. Vascular endothelial dysfunction is characterised by altered permeability barrier function, enhanced adhesion molecule expression, increased leukocyte adhesion, impaired endothelium-dependent vasodilator responses, enhanced thrombosis, impaired fibrinolysis, a reduction in endogenous nitric oxide activity, reduced vascular smooth muscle cell function and increased arterial stiffness [4]. Two measures of vascular function that have been used to predict CVD in the general population include carotid intima-media thickness (cIMT), a non-invasive ultrasound biomarker of early atherosclerosis [5] and carotid-femoral pulse wave velocity (PWV), a measure of arterial compliance as well as stiffness [6].

Diet has long been considered to play a role in the development of CVD and the link between diet and vascular health is thought to be mediated through multiple biological pathways [7], [8]. Diet, including entire diets (the Mediterranean diet), individual dietary components (e.g. fish-oil and fruit and vegetables), or diet-related genetic polymorphisms, have been shown to be associated with vascular function outcomes [9]. A mechanism through which diet may affect vascular health is through its influence on homocysteine (Hcy) concentrations. Increased homocysteine (Hcy) concentrations have been associated with an increased CVD risk [10], [11], [12]. Hcy concentrations are closely linked with vitamin B-12 and folate, where deficiencies in these nutrients can result in disruptions to the methylation pathway causing elevated Hcy concentrations [13]. Supplementation with these nutrients, particularly folate, has been shown to reduce Hcy concentrations [14], [15]. Therefore it is plausible that a diet consisting of high intakes of folate and vitamin B12, in turn characterised by low Hcy concentrations may have a positive effect on vascular health. Observational studies demonstrate a beneficial association between B-vitamins and CVD but large clinical trials have not been supportive of this [11]. However, baseline levels of B-vitamins and Hcy in these trials may not have been sufficiently low to observe a beneficial effect of supplementation, and these studies have mainly focused on secondary prevention with results from smaller trials suggesting the role of B-vitamins in preventing atherogenesis and other adverse vascular conditions remains plausible [11], [16].

While studies investigating individual associations between Hcy, B-vitamins and vascular health can provide valuable insight, examining dietary patterns (DPs) that capture a broader picture of food and nutrients may be more predictive of disease risk than individual nutrients [17]. One way of investigating the association between DPs and early markers of vascular function via its influence on Hcy and B-vitamins is through the statistical method, reduced rank regression (RRR) [18]. RRR is a hypothesis-based empirical method that uses prior knowledge of a specific pathway to identify DPs [18]. To our knowledge, no previous study has examined a DP characterised by folate intake, vitamin B12 intake and plasma Hcy and its relationship with vascular function as an early marker of CVD risk. We aimed to use longitudinal data from a cohort of British adults to 1) identify a DP characterised by dietary folate intake, vitamin B12 intake and Hcy concentrations using RRR and, 2) examine its long-term relationships with two markers of vascular function.

## 2 Methods

### 2.1 Participants

**Fig. 1:**
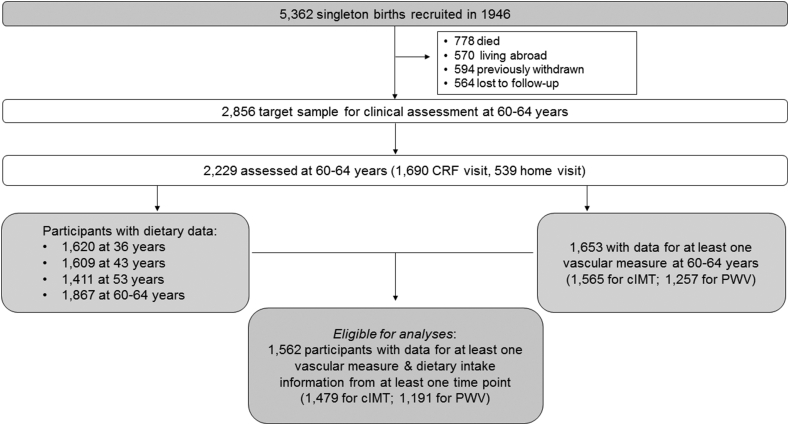
Number of participants in the MRC National Survey of Health and Development and selection for the present study. CRF: clinical research facility; cIMT: carotid intima-media thickness; PWV: pulse wave velocity.

The MRC National Survey of Health and Development (NSHD) consists of 5362 singleton births from married parents in England, Scotland and Wales, stratified by social class [19]. The cohort has been followed up 24 times since birth [20]. In 2006–2010 (60–64 years), eligible study members were invited for an assessment at one of six clinical research facilities (CRF) or for home visits by a trained research nurse. Invitations were not sent to those who had died (*n* = 778), who were living abroad (*n* = 570), had previously withdrawn from the study (*n* = 594), or had been lost to follow-up (*n* = 564). Of the responders to the 60–64 year follow-up, individuals attending the CRFs (*n* = 1690) had lower adiposity and lifetime smoking exposure and higher levels of physical activity compared with those who did not attend the CRF [21]. Eligible participants for the main analyses included those with at least one vascular measure at 60–64 years and who provided information on three or more days of dietary intake from at least one adult time point (*n* = 1562) (Fig. 1). Ethical approval was obtained from the Greater Manchester and the Scotland Research Ethics Committees and participants provided written and informed consent.

### 2.2 Primary outcomes: vascular measures at 60–64 years

Vascular measurements were taken by trained research staff during the CRF visits following an overnight fast and prior to any strenuous physical activity. cIMT was measured in the left and right common carotid artery from three images taken in the lateral view on each side using high resolution B-mode ultra-sound imaging (Vivid I [12 MHz probe]; GE Healthcare) [22]. Participants with carotid plaque i.e. cIMT greater than 1.5 mm, with abnormal shape and abnormal wall texture were excluded (*n* = 13). Further details of how cIMT was measured are outlined in previous papers [23]. A combined average of left and right cIMT is used in analyses.

PWV was calculated using pulse pressure waveforms obtained from the carotid and femoral arteries using the Vicorder device (Skidmore Medical). A 100 mm wide pressure cuff was placed at the upper right thigh and another 30 mm partial cuff was placed directly over the right carotid artery. Cuffs were inflated simultaneously to 65 mmHg for approximately 10–15 s. Path length was measured between the cuffs and defined as the distance between the suprasternal notch directly to the top of the femoral cuff. PWV was automatically calculated by an in built algorithm.

### 2.3 Secondary outcomes: CVD-risk factors at 60–64 years

We considered blood pressure, blood lipids, triglycerides, and C-reactive protein (CRP) measured in overnight fasting blood samples at 60–64 years as secondary outcomes [24]. Details of the assay used to estimate low density lipoprotein (LDL), high density lipoprotein (HDL) and total cholesterol, triglycerides and CRP have previously been published [23], [25]. Blood pressure was measured twice using an Omron HEM-705 with the participant in a seated position, with the second reading used for this analysis. Information on medication use was collected and coded according to the British National Formulary, from which individuals on lipid lowering and antihypertensive medications were identified.

### 2.4 Dietary assessment in adulthood

Dietary data were collected when participants were aged 36, 43, 53 and 60–64 years [26], [27], [28], [29]. All food and drink consumed both at home and away were recorded by participants in a 5-day estimated diet diary [30]. Detailed guidance notes and photographs were provided to assist participants [28]. Dietary intake was coded in the MRC Human Nutrition Research, Cambridge using the in-house programmes, Diet In Data Out (DIDO) and Diet In Nutrients Out (DINO) [26], [27]. Nutrient intakes were calculated based on McCance and Widdowson's *The Composition of Foods* taking into account food composition, fortification and portion sizes [29]. We aggregated intakes of individual foods and drinks into 46 food groups (g/day) based on nutrient profile and culinary usage (Supplementary Table 1).

We used an individualised method to identify dietary misreporting. This involved assigning a Physical Activity Level (PAL) to each individual based on self-reported responses to questions on leisure time physical activity and calculating the ratio of estimated intake (EI) to estimated energy requirement (EER) and its 95% confidence interval (CI) for each year [31], [32], [33]. Individuals were grouped into underreporters (EI:EER <95 CI EI:EER), plausible reporters (EI:EER within the 95% CI) or overreporters (EI:EER >95%CI EI:EER), coded as 0, 1 and 2, respectively. A cumulative dietary misreporting variable across adulthood was created by combining responses to the 4 time points where 0 indicates underreporting at all time points and 8 indicates overreporting at all time points.

### 2.5 Intermediate variables for RRR

We considered three variables as the intermediate variables in RRR analyses: plasma Hcy, dietary intake of folate and dietary intake of vitamin B-12. Total plasma Hcy (μmol/l) was determined from participant's samples at 60–64 years using a LTQ Orbitrap Elite mass spectrometer coupled to an Ultimate 3000 ultra high performance liquid chromatography (Thermo Fisher) in the Department of Biochemistry University of Cambridge. The Orbitrap was operated over a mass range of 60–1500 m/z at a resolution of 3000 ppm. Data were integrated in Xcalibur (Thermo Fisher) and the homocysteine signal (μmol/l) was normalised to the deuterated internal standard. We obtained dietary folate (μg/1000 kcal) and vitamin B12 (μg/1000 kcal) as nutrient densities from the diet diaries.

### 2.6 Covariates in adulthood

We identified relevant covariates *a priori*. These included: socioeconomic position (SEP), body mass index (BMI), smoking status, physical activity and presence of diabetes.

Since some participants had retired by 60–64 years, SEP was based on occupational social class at age 53 years (or 43 years if missing (*n* = 112)), grouped into categories according to the UK Registrar General's classification (professional and intermediate; skilled non-manual; skilled manual; semi-skilled and unskilled manual).

Height and weight were measured at 60–64 years. BMI was calculated as weight (kg) divided by height (m^2^).

Smoking status was categorised as never, current or ex-smoker at 60–64 years (or 53 or 69 years if missing (*n* = 115)).

Accumulation of leisure time physical activity (LTPA) across adulthood was derived as previously described [34]. At each age, we categorised participants as inactive, moderately active or most active. The overall score is the sum of responses at each time point, ranging from 0 (inactive) to 8 (active). LTPA across adulthood was categorised as always inactive, active in at least one time point, active at more than one time point.

Self-report of doctor diagnosis of diabetes (type 1 or type 2) up to 60–64 years was categorised as yes/no.

### 2.7 Statistical analyses

#### 2.7.1 Reduced rank regression

We used reduced rank regression (RRR) to identify a DP characterised by plasma Hcy, dietary folate, and dietary vitamin B12, using data from participants at 60–64 years. RRR DPs are weighted linear combinations of food intake that explain the maximum variation in a set of pre-defined intermediate variables i.e. variables that are hypothesised to be on the pathway between food intake and the outcome of interest. We applied RRR to all 1867 participants with dietary data (regardless of whether they had vascular measures or not) at 60–64 years using the 46 food groups (g/day) as predictors and plasma Hcy (μmol/l), dietary folate (μg/1000 kcal) and dietary vitamin B12 (μg/1000 kcal) as intermediate variables. Since there were no major differences between the DPs of men and women, the presented DP is based on data from men and women combined.

For each completed food diary, the participant received a z-score. This z-score represents the degree to which the participant's reported intake reflected the DP obtained from RRR in relation to other individuals in the study (mean = 0, SD = 1).

#### 2.7.2 Confirmatory reduced rank regression

Following identification of the DP at 60–64 years, we estimated DP z-scores at the three younger ages. A total of 2107 participants had dietary information from one or more time points. It was not possible to independently identify the DP at younger ages since Hcy concentrations were available at 60–64 years only. Therefore to score participants in relation to DP at earlier ages, we applied confirmatory RRR using the DP identified in the 60–64 year follow-up [35]. This involved using the scoring weights produced by the 60–64 year RRR DP and applying them to reported dietary intakes from earlier years. This results in each participant receiving a z-score which quantifies how much their reported dietary intake at each time point reflects the DP identified at 60–64 years. To assess long-term adherence to the identified DP, we calculated an average DP z-score for each individual with DP z-scores from at least 2 time points.

### 2.8 Primary analyses

Since the distribution of DP z-scores was found to vary by sex, we grouped DP z-scores into fifths using sex-specific cut-points. We assessed differences in selected nutrient intakes and intermediate variables according to sex-specific DP fifths at 60–64 years using linear regression models.

To enhance comparability between the outcomes, we standardised cIMT and PWV for use in subsequent regression models. We assessed associations between sex-specific groups of the DP z-scores (with the lowest fifth, Q1, as reference) at each time point and vascular function at 60–64 years separately using a series of linear regression models. The regression models were adjusted for: 1) participant characteristics (dietary misreporting and socioeconomic position) 2) lifestyle factors (BMI, smoking and physical activity) 3) diabetes. Interactions between the DP and sex and the DP and smoking were assessed. We examined non-linear associations between the DP z-scores and vascular outcomes using a likelihood ratio test comparing models with the DP groups fitted as a linear term to a model with the DP groups fitted as a categorical term.

We applied linear regression models with the same adjustments outlined above to investigate average DP z-scores over time and vascular outcomes.

### 2.9 Sensitivity analyses

In sensitivity analyses, we additionally adjusted associations between the DP and vascular function for the use of folic acid or multivitamin dietary supplements, as well as lipid lowering and/or antihypertensive medication. Information for folic acid supplementation was available for participants at 60–64 years only and there was no information on the use of any nutrient supplementation at 43 years.

### 2.10 Secondary analyses

In secondary analyses, we investigated whether adherence to the identified DP was associated with other CVD-risk factors using a similar series of regression models. We applied censored regression where use of antihypertensive/lipid-lowering medication were likely to affect the level of the outcome (i.e. blood pressure, cholesterol and triglycerides) [36].

### 2.11 Multiple imputation

Of the eligible participants (*n* = 1562), 97%, had complete data for all covariates. Missing values were most frequent for SEP (3%) followed by smoking status (1%). To account for missing data on covariates used in regression models, we performed multiple imputation by chained equations where twenty complete datasets were created [37]. The results based on regression analyses using multiple imputation were similar to those conducted on complete cases, but with slightly more precise standard errors. The imputed results are presented.

We performed RRR using the PROC PLS procedure in SAS and all other analyses were performed using STATA version 14.

## 3 Results

### 3.1 Participant characteristics

Table 1 displays demographics, lifestyle factors and vascular phenotypes of the 1562 participants with relevant data. Means for cIMT and PWV were slightly higher for men, among those with a higher BMI, with diabetes, who smoked (for cIMT only), and reported taking either blood-pressure or lipid-lowering medication (*p* ≤ 0.008).

**Table 1:** Characteristics of participants with at least one vascular measure and dietary data from at least one time point (n = 1562).

	Total		Male (*N* = 743)		Female (*N* = 819)	
	N	% or mean (SD)	N	% or mean (SD)	N	% or mean (SD)
***Demographics***						
*Socioeconomic position at 53 years (or 43 years if missing)*						
Professional & intermediate	816	52.2	459	61.78	357	43.59
Skilled non-manual	363	23.2	80	10.77	283	34.55
Skilled Manual	193	12.4	144	19.38	49	5.98
Semi-skilled and unskilled manual	151	9.7	50	6.73	101	12.33
Missing	39	2.5	10	1.4	29	3.5
*P* sex difference^b^	<0.001					
***Lifestyle-related factors***						
*BMI at 60–64 years, kg/m*^*2*^	1562	27.5 (4.6)	743	27.6 (3.8)	819	27.5 (5.1)
Missing	0					
*P* sex difference^b^	0.40					
*Self-reported physical activity since 1982*						
Inactive	199	12.7	85	11.44	114	13.92
Active at some point	1272	81.4	605	81.43	667	81.44
Always active	90	5.8	53	7.13	37	4.52
Missing	1	0.1	0	0	1	0.1
*P* sex difference^b^	0.06					
*Smoking status*						
Current	167	10.7	82	11.04	85	10.38
Ex-smoker	873	55.9	442	59.49	431	52.63
Never smoker	506	32.4	209	28.13	297	36.26
Missing	16	1.0	10	1.4	6	0.7
*P* sex difference^b^	0.005					
***Medical conditions at 60–64 years***						
*Anti-hypertensive medication*						
No	1097	70.2	503	67.7	594	72.53
Yes	419	26.8	213	28.67	206	25.15
Missing	46	2.9	27	3.6	19	2.3
*P* sex difference^b^	0.07					
*Lipid lowering medication*						
No	1236	79.1	550	74.02	686	83.76
Yes	326	20.9	193	25.98	133	16.24
Missing	0					
*P* sex difference^b^	<0.001					
*Diabetes*						
No	1477	94.6	701	94.35	776	94.75
Yes	83	5.3	42	5.65	41	5.01
Missing	2	0.1	0	0	2	0.24
*P* sex difference^b^	0.35					
***Vascular phenotype at 60–64 years***						
*Pulse wave velocity, m/s*	1191	8.18 (1.5)	551	8.35 (1.44)	640	8.04 (1.56)
Missing	371		192		179	
*P* sex difference^b^	<0.001					
*Carotid intima-media thickness, mm*	1479	0.69 (0.13)	707	0.71 (0.14)	772	0.67 (0.11)
Missing	83		36		47	
*P* sex difference^b^	<0.001					
*Systolic blood pressure*	1557	135.61 (18.16)	742	138.98 (18.03)	815	135.53 (17.74)
Missing	5		1		4	
*P* sex difference^b^	<0.001					
*Diastolic blood pressure*	1558	77.21 (9.82)	743	78.88 (9.88)	815	75.69 (9.51)
Missing	4		0		4	
*P* sex difference^b^	<0.001					
*Total cholesterol, mmol/L*	1492	5.69 (1.19)	718	5.32 (1.08)	774	6.03 (1.18)
Missing	70		25		45	
*P* sex difference^b^	<0.001					
*Low density lipoprotein cholesterol*	1452	3.53 (1.02)	692	3.30 (0.94)	760	3.74 (1.05)
Missing	110		51		59	
*P* sex difference^b^	<0.001					
*High density lipoprotein cholesterol*	1492	1.60 (0.40)	718	1.41 (0.33)	774	1.76 (0.40)
Missing	70		25		45	
*P* gender difference^b^	<0.001					
*Triglycerides, mmol/L*	1460	1.12 (1.09, 1.14)^a^	698	1.23 (1.18, 1.27)	762	1.03 (0.99, 1.06)
Missing	102		45		57	
*P* sex difference^b^	<0.001					
*C-reactive protein, mg/L*	1491	2.18 (2.08, 2.28)^a^	718	2.12 (1.99, 2.26)	773	2.23 (2.10, 2.37)
Missing	71		25		46	
*P* sex difference^b^	0.21					

a Values are geometric mean (95% CI).

b χ^2^ for categorical variables; t-test/Wilcoxon-rank sum for continuous variables.

Participants with data for at least one vascular outcome and information on DP scores from at least one time point (*n* = 1562) were more likely to be of higher SEP, had a lower mean BMI, lower lipids, cholesterol, blood pressure and CRP values and had generally healthier lifestyles than those responding to the 60–64 year follow-up who did not have vascular function information (*n* = 667, Supplementary Table 2).

### 3.2 Dietary pattern

Of the 2107 participants with dietary information from one or more time points 77% had dietary information for the first time at 36y. A total of 73%, 76%, 64% and 65% participants were identified as plausible dietary reporters at 36, 43, 53 and 60–64 years respectively.

The three intermediate variables (plasma Hcy, dietary folate and dietary vitamin B12) were not found to be highly correlated with each other (*r* ≤ 0.02 for all pairwise correlations). Since three intermediate variables were used in RRR models, three DPs were identified.

The first DP identified was strongly and positivity correlated with dietary folate, had a moderate positive correlation with dietary vitamin B12 and was weakly correlated with plasma Hcy (Supplementary Table 3). The first DP, which will be referred to as the high B-vitamin DP, explained 4.5% of the variation in all three intermediate variables. The second and third DPs explained considerably less variation in intermediate variables: 1.7% and 0.88% respectively (Supplementary Table 3). Since the first high B-vitamin DP explained the highest percent of the variation in intermediate variables, only this DP was taken forward for subsequent analysis.

**Fig. 2:**
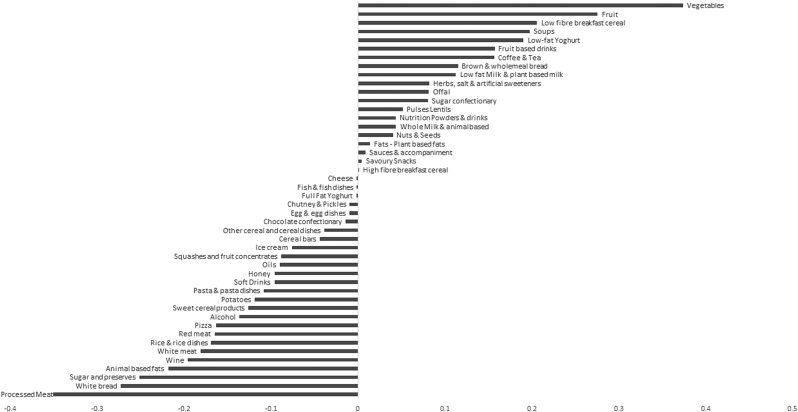
Factor loadings for the high B-vitamin dietary pattern at 60–64 years.

A higher DP z-score for the high B-vitamin DP was characterised by higher intakes of vegetables, fruit, and low fibre breakfast cereals, and low intakes of processed meat, white bread and, sugar and preserves (Fig. 2). Details of the second and third DPs obtained from these analyses are outlined in Supplementary Figs. 1 and 2 and Supplementary Table 3.

**Fig. 3:**
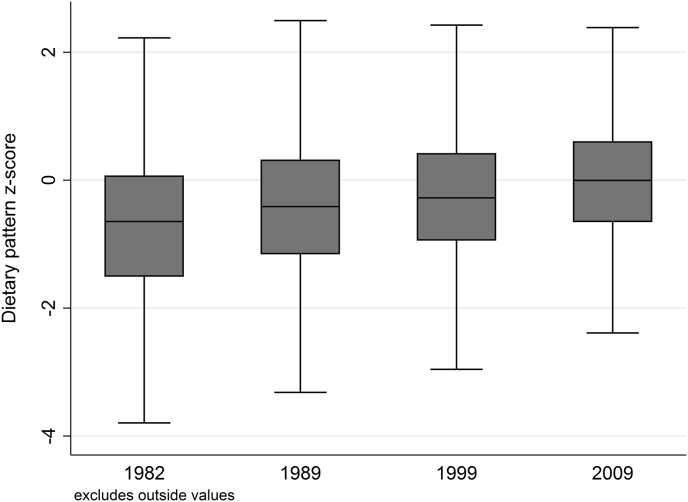
High B-vitamin dietary pattern z-scores over time.

Dietary intakes of folate and vitamin B12 were highest in the top fifth of the DP distribution. However, Hcy concentrations did not change according to z-scores for the high B-vitamin DP (Table 2). In addition, total energy intakes and percentage energy from fat decreased, while percentage energy from carbohydrate and protein increased with increasing DP fifths (Table 2). However, the size of the difference in these nutrients across groups was small (Table 2). Mean z-scores for the high B-vitamin DP did not change extensively over time (see Fig. 3).

**Table 2:** Characteristics of high B-vitamin dietary pattern by sex-specific dietary pattern quintile at 60–64 years (*n* = 1867).

	Total (*n* = 1867)		DP Q1 (*n* = 374)		DP Q2 (*n* = 373)		DP Q3 (*n* = 373)		DP Q4 (*n* = 374)		DP Q5 (*n* = 373)		*P*_trend_
Dietary pattern score	−0.01	(1.05)	−1.35	(0.67)	−0.49	(0.35)	−0.02	(0.31)	0.45	(0.30)	1.36	(0.82)	<0.001
Plausible reporters (%)	66.80		76.20		65.15		67.56		64.97		60.05		
***Intermediate variables***													
Homocysteine (μmol/l)	9.79	(6.87)	9.57	(6.58)	9.56	(7.03)	9.43	(6.41)	10.46	(7.29)	9.91	(7.02)	0.20
Dietary Folate (μg/d)	348.93	(212.74)	299.80	(110.01)	321.91	(130.32)	335.84	(120.51)	349.49	(132.34)	437.74	(393.16)	<0.001
Dietary Vitamin B12 (μg/d)	8.97	(30.46)	6.36	(3.89)	8.28	(26.92)	8.42	(18.71)	8.22	(21.38)	13.59	(55.47)	0.004
***Nutrients***													
Energy (kcal/d)	1879.12	(458.53)	2046.70	(451.11)	1925.69	(448.49)	1847.49	(430.57)	1798.26	(445.45)	1777.23	(465.52)	<0.001
% Energy from Fat	33.97	(5.88)	35.36	(6.08)	34.57	(5.67)	33.87	(5.68)	33.43	(5.28)	32.62	(6.31)	<0.001
% Energy from CHO	46.88	(7.34)	44.95	(7.76)	46.03	(7.11)	47.05	(6.52)	47.19	(7.32)	49.17	(7.30)	<0.001
% Energy from Protein	16.68	(3.07)	15.91	(2.80)	16.21	(2.67)	16.60	(2.75)	17.13	(3.25)	17.58	(3.51)	<0.001
***Top positive loading food groups (*g/d*)***													
Vegetables	171.97	(91.56)	126.49	(63.76)	149.17	(72.16)	164.52	(74.24)	184.65	(83.02)	235.17	(116.36)	<0.001
Fruit	152.85	(114.86)	111.82	(97.56)	130.05	(102.36)	149.89	(110.17)	165.70	(110.69)	206.92	(128.15)	<0.001
Low fibre breakfast cereal	5.92	(15.82)	2.25	(6.46)	4.16	(8.18)	4.56	(9.03)	6.08	(10.06)	12.59	(30.01)	<0.001
***Top negative loading food groups (*g/d*)***													
Processed meats	32.19	(31.40)	47.28	(42.88)	36.22	(29.22)	30.28	(27.30)	26.12	(24.78)	21.01	(21.81)	<0.001
White bread	45.18	(41.32)	63.04	(46.95)	50.32	(41.92)	45.58	(38.41)	38.03	(37.45)	28.91	(32.46)	<0.001
Sugar and preserves	11.64	(15.12)	17.47	(19.40)	13.03	(16.12)	10.97	(13.32)	9.56	(12.88)	7.17	(10.26)	<0.001

Values are mean (SD) unless otherwise indicated.

CHO: Carbohydrate.

### 3.3 Associations between the high B-vitamin DP z-scores and vascular function

Although participants in the top fifth of the DP distribution at 36 years had lower cIMT compared with those in the bottom fifth (p_trend_ = 0.09), this attenuated slightly following adjustment for lifestyle factors and diabetes (Table 3). There was no evidence of a linear trend or deviation from linearity in any model (Table 3, Table 4). There was no evidence for interaction between the DP and sex or the DP and smoking (*p*_interaction_ ≥ 0.05 for all ages). Additional adjustment for the use of folic acid or multivitamin dietary supplements, or use of lipid lowering and/or antihypertensive medication did not alter the estimates presented.

**Table 3:** Association between the high B-vitamin dietary pattern sex-specific quintiles and carotid intima media thickness.^a^

	DP Q1		DP Q2		DP Q3		DP Q4		DP Q5		*P*_trend_
			Coef	(95% CI)	Coef	(95% CI)	Coef	(95% CI)	Coef	(95% CI)	
***36 years n = 1137***											
Model 1		Ref	−0.10	(−0.30, 0.09)	−0.20	(−0.40, −0.01)	−0.06	(−0.26, 0.13)	−0.22	(−0.42, −0.02)	0.09
Model 2		Ref	−0.11	(−0.30, 0.08)	−0.20	(−0.40, −0.01)	−0.06	(−0.25, 0.14)	−0.21	(−0.41, −0.01)	0.12
Model 3		Ref	−0.11	(−0.31, 0.08)	−0.20	(−0.40, −0.01)	−0.06	(−0.26, 0.14)	−0.21	(−0.41, −0.02)	0.11
***43 years n = 1152***											
Model 1		Ref	−0.08	(−0.27, 0.11)	0.01	(−0.19, 0.20)	−0.08	(−0.28, 0.11)	−0.14	(−0.34, 0.06)	0.21
Model 2		Ref	−0.06	(−0.25, 0.12)	0.03	(−0.17, 0.22)	−0.05	(−0.24, 0.15)	−0.09	(−0.29, 0.10)	0.45
Model 3		Ref	−0.06	(−0.25, 0.12)	0.02	(−0.17, 0.22)	−0.05	(−0.25, 0.14)	−0.09	(−0.29, 0.10)	0.43
***53 years n = 1007***											
Model 1		Ref	−0.19	(−0.39, 0.02)	−0.08	(−0.29, 0.12)	−0.18	(−0.39, 0.02)	−0.05	(−0.25, 0.15)	0.78
Model 2		Ref	−0.17	(−0.38, 0.03)	−0.04	(−0.25, 0.16)	−0.15	(−0.35, 0.06)	−0.004	(−0.20, 0.20)	0.83
Model 3		Ref	−0.17	(−0.37, 0.03)	−0.04	(−0.24, 0.17)	−0.14	(−0.35, 0.06)	−0.01	(−0.21, 0.19)	0.88
***60–64 years n = 1352***											
Model 1		Ref	−0.05	(−0.23, 0.12)	−0.10	(−0.27, 0.07)	−0.13	(−0.30, 0.04)	−0.04	(−0.21, 0.13)	0.42
Model 2		Ref	−0.05	(−0.22, 0.12)	−0.08	(−0.25, 0.09)	−0.10	(−0.27, 0.07)	−0.01	(−0.18, 0.16)	0.79
Model 3		Ref	−0.05	(−0.22, 0.13)	−0.08	(−0.25, 0.09)	−0.10	(−0.27, 0.07)	−0.01	(−0.18, 0.16)	0.71

Model 1: dietary misreporting, socioeconomic status.

Model 2: dietary misreporting, socioeconomic status, BMI, smoking status, physical activity.

Model 3: dietary misreporting, socioeconomic status, BMI, smoking status, physical activity, diabetes.

a Imputed covariates, standardised outcome.

**Table 4:** Association between the high B-vitamin dietary pattern sex-specific quintiles and pulse wave velocity.^a^

	DP Q1	DP Q2		DP Q3		DP Q4		DP Q5		*P*_trend_
		Coef	(95% CI)	Coef	(95% CI)	Coef	(95% CI)	Coef	(95% CI)	
***36 years n = 920***										
Model 1	Ref	−0.34	(−0.57, −0.12)	−0.01	(−0.23, 0.21)	−0.04	(−0.26, 0.18)	0.02	(−0.20, 0.24)	0.14
Model 2	Ref	−0.35	(−0.58, −0.13)	−0.03	(−0.25, 0.19)	−0.05	(−0.27, 0.17)	0.001	(−0.22, 0.22)	0.17
Model 3	Ref	−0.36	(−0.58, −0.13)	−0.03	(−0.25, 0.19)	−0.05	(−0.27, 0.18)	0.001	(−0.22, 0.22)	0.17
***43 years n = 937***										
Model 1	Ref	0.08	(−0.12, 0.28)	−0.08	(−0.29, 0.13)	−0.05	(−0.26, 0.16)	0.10	(−0.11, 0.31)	0.76
Model 2	Ref	0.08	(−0.12, 0.28)	−0.07	(−0.28, 0.14)	−0.03	(−0.24, 0.18)	0.13	(−0.08, 0.34)	0.56
Model 3	Ref	0.08	(−0.12, 0.28)	−0.08	(−0.29, 0.13)	−0.03	(−0.24, 0.18)	0.13	(−0.09, 0.34)	0.57
***53 years n = 826***										
Model 1	Ref	0.05	(−0.17, 0.27)	0.11	(−0.11, 0.33)	−0.12	(−0.34, 0.10)	−0.06	(−0.28, 0.15)	0.22
Model 2	Ref	0.07	(−0.15, 0.29)	0.14	(−0.08, 0.36)	−0.09	(−0.31, 0.13)	−0.04	(−0.25, 0.18)	0.32
Model 3	Ref	0.08	(−0.14, 0.30)	0.15	(−0.07, 0.37)	−0.09	(−0.31, 0.13)	−0.04	(−0.25, 0.18)	0.32
***60–64 years n = 1092***										
Model 1	Ref	−0.15	(−0.34, 0.04)	−0.06	(−0.25, 0.13)	−0.08	(−0.27, 0.11)	0.06	(−0.13, 0.24)	0.33
Model 2	Ref	−0.15	(−0.34, 0.04)	−0.05	(−0.24, 0.13)	−0.07	(−0.26, 0.12)	0.07	(−0.11, 0.26)	0.25
Model 3	Ref	−0.15	(−0.34, 0.04)	−0.05	(−0.24, 0.14)	−0.07	(−0.26, 0.12)	0.07	(−0.12, 0.26)	0.27

Model 1: dietary misreporting, socioeconomic status.

Model 2: dietary misreporting, socioeconomic status, BMI, smoking status, physical activity.

Model 3: dietary misreporting, socioeconomic status, BMI, smoking status, physical activity, diabetes.

a Imputed covariates, standardised outcome.

### 3.4 Associations between average z-score for the high B-vitamin DP and vascular function

Compared with the bottom fifth, participants in higher average DP groups had lower cIMT scores. However the association attenuated following adjustment for lifestyle factors. There was no evidence for an association between average high B-vitamin DP z-score and cIMT or PWV (Table 5).

**Table 5:** Association between long-term adherence to the high B-dietary pattern sex-specific quintiles and vascular function.^a^

	DP Q1	DP Q2		DP Q3		DP Q4		DP Q5		*P*_trend_
		Coef	(95% CI)	Coef	(95% CI)	Coef	(95% CI)	Coef	(95% CI)	
***Carotid intima media thickness n = 1335***										
Model 1	Ref	−0.08	(−0.28, 0.11)	−0.24	(−0.43, −0.05)	−0.21	(−0.40, −0.02)	−0.17	(−0.37, 0.02)	0.05
Model 2	Ref	−0.08	(−0.28, 0.11)	−0.22	(−0.41, −0.02)	−0.18	(−0.37, 0.01)	−0.12	(−0.32, 0.07)	0.18
Model 3	Ref	−0.09	(−0.29, 0.10)	−0.22	(−0.41, −0.03)	−0.19	(−0.38, 0.01)	−0.14	(−0.33, 0.06)	0.16
**Pulse wave velocity *n* = 1081**										
Model 1	Ref	0.01	(−0.20, 0.22)	−0.10	(−0.32, 0.11)	−0.003	(−0.21, 0.21)	0.05	(−0.16, 0.27)	0.60
Model 2	Ref	0.003	(−0.21, 0.22)	−0.10	(−0.31, 0.12)	−0.004	(−0.22, 0.21)	0.06	(−0.15, 0.28)	0.50
Model 3	Ref	0.001	(−0.21, 0.22)	−0.10	(−0.31, 0.12)	−0.004	(−0.22, 0.21)	0.06	(−0.15, 0.28)	0.51

Model 1: dietary misreporting, socioeconomic status.

Model 2: dietary misreporting, socioeconomic status, BMI, smoking status, physical activity.

Model 3: dietary misreporting, socioeconomic status, BMI, smoking status, physical activity, diabetes.

a Imputed covariates, standardised outcome, restricted to those with information from ≥2 years of dietary intake.

### 3.5 Associations between average z-score for the high B-vitamin DP and other CVD risk factors

There was evidence for an inverse association between average DP z-scores and CRP, and triglycerides (Table 6). There was no evidence for an association between the high B-vitamin DP and any of the other CVD risk factors examined (Table 6).

**Table 6:** Association between long-term adherence to the high B-dietary pattern sex-specific quintiles and additional CVD risk factors.^a^^,^^b^

	N	DP Q1	DP Q2		DP Q3		DP Q4		DP Q5		*P*_trend_
			Coef	(95% CI)	Coef	(95% CI)	Coef	(95% CI)	Coef	(95% CI)	
Systolic blood pressure (mmHg) at 60–64 years^c^	1371	Ref	0.14	(−3.91, 4.18)	−2.79	(−6.68, 1.10)	−1.04	(−4.99, 2.90)	−0.32	(−4.40, 3.76)	0.76
Diastolic blood pressure (mmHg) at 60–64 years^c^	1372	Ref	1.14	(−1.03, 3.32)	−1.06	(−3.15, 1.03)	0.48	(−1.64, 2.60)	0.93	(−1.27, 3.12)	0.61
Total cholesterol (mmol/L) at 60–64 years^d^	1345	Ref	−0.03	(−0.25, 0.18)	−0.03	(−0.23, 0.18)	0.02	(−0.19, 0.23)	−0.12	(−0.34, 0.10)	0.45
LDL cholesterol (mmol/L) at 60–64 years^d^	1311	Ref	−0.08	(−0.26, 0.11)	−0.03	(−0.21, 0.15)	−0.002	(−0.19, 0.18)	−0.11	(−0.30, 0.08)	0.56
HDL cholesterol (mmol/L) at 60–64 years	1345	Ref	0.01	(−0.07, 0.08)	0.02	(−0.05, 0.10)	0.04	(−0.04, 0.11)	0.05	(−0.03, 0.12)	0.15
Triglycerides (ln(mmol/L)) at 60–64 years^d^	1318	Ref	4.06	(−6.86, 14.98)	−6.58	(−17.05, 3.88)	−2.18	(−12.91, 8.54)	−11.81	(−22.81, −0.82)	0.01
C-reactive protein (ln(mg/L)) at 60–64 years	1345	Ref	−10.87	(−27.22, 5.47)	−21.71	(−37.48, −5.94)	−8.99	(−25.04, 7.07)	−25.73	(−42.22, −9.25)	0.01

a Includes eligible sample with dietary information from ≥2 time points.

b Adjusted for dietary misreporting.

c Censored regression, values censored for anti-hypertensive medication use.

d Censored regression, values censored for lipid lowering medication use.

## 4 Discussion

In this British birth cohort, we identified a high B-vitamin DP consisting of high intakes of vegetables and fruit and low intakes of processed meat and white bread. There was no consistent evidence for an association between scores for this DP at any age and cIMT or PWV. There was an association between the high B-vitamin DP and CRP and triglycerides suggesting that the high B-vitamin DP may impact the development of CVD through pathways described by novel markers of vascular function, including inflammation. These markers provide information on vascular/endothelial dysfunction, thought to represent an integrated CVD risk pathway.

The main strength of the study is the use of a nationally representative sample containing repeated dietary measures over a 30 year period which enabled prospective investigations at various time points. Despite some attrition, this cohort remained broadly representative of the white British population born in the early post world war II period [19], [21]. One novel aspect of this study was the examination of two vascular function markers, cIMT and PWV. This supports examination of the relationship between diet and CVD at an earlier stage of aetiopathogenesis. Another novel aspect was use of RRR, with intermediate variables from both plasma measurements and dietary intake to define the DP. Unfortunately, Hcy concentrations were only available at one time point, therefore confirmatory RRR had to be applied using weights from the 60–64 year follow-up to examine the DP at the previous time points. This approach has been used in previous studies [35], [38]. While it is possible that different DPs may have emerged at the earlier years, our approach has the advantage of assessing adherence to the same defined DP over time. While the measurement of dietary intake is not without error and may be prone to bias [33], the identification of individual level dietary misreporting in this study attempted to control for this.

Few studies have examined associations between DPs and vascular function assessed by cIMT and PWV. Findings from studies examining the relationship between DPs derived using principal component analysis (PCA) and PWV or cIMT have been equivocal and difficult to compare due to the range of DPs identified [39], [40], [41], [42]. Our observed RRR-derived high B-vitamin DP consisted of foods (vegetables, fruit and fortified foods including breakfast cereals) recognised as major sources of dietary folate in the UK population [43] while food groups that are sources of vitamin B12, such as meat and fish, did not have strong factor loadings on this pattern. Although, we observed an increase in folate and B12 intakes in the highest fifth of the high B-vitamin DP distribution, there was no change in Hcy concentrations. Increased Hcy concentrations have been associated with risk of CVD [12], and dietary modifications have been shown to effect Hcy concentrations [44], [45]. Previous studies have demonstrated an inverse association between Hcy concentrations and the B-vitamins, folate and vitamin B12 [46], [47], suggesting that diets high in B-vitamins may be protective against CVD via its influence on Hcy concentrations. Results from a 4-week intervention trial found that increased consumption of vegetables and citrus fruits, both good sources of folate, had the dual effects of improving folate status, while also decreasing Hcy concentrations [48]. However in our study, folate and vitamin B12 intakes and plasma Hcy concentrations were not correlated. The majority of participants (92.4% and 99.6%) at 60–64 years had folate and vitamin B12 intakes above the UK recommended nutrient intakes [49]. Therefore any correlation between these nutrients and Hcy may be reduced.

To our knowledge, only one other study used RRR to identify DPs that were characterised by Hcy, folate and vitamin B12 [50]. In the Coronary Risk Factors for Atherosclerosis in Women (CORA) study, DPs were created using data from 455 women (200 coronary heart disease cases; 255 controls). The DP identified was high in wholegrain bread, fresh fruit, olive oil, mushrooms, cruciferous vegetables, wine and nuts and low in fried potatoes, and was positively associated with folate and B12, but negatively associated with Hcy. The authors observed a reduced risk for coronary heart disease with increasing scores for the DP in both the CORA study and prospectively in the European Prospective Investigation into Cancer and Nutrition (EPIC)-Potsdam Study. In comparison with our study, the authors used plasma concentrations of all three intermediate variables, Hcy, folate and vitamin B-12, and risk of coronary heart disease was the main outcome. Their DP explained 8.9% of the variation in all three biomarkers, whereas our DP explained 4.5% of the variation shared by plasma Hcy, dietary folate and vitamin B-12.

In the Multi-Ethnic Study of Atherosclerosis study, authors used RRR to derive a DP with inflammatory markers (CRP, interleukin-6, Hcy and fibrinogen) as intermediate variables [41]. The authors identified an inverse association between higher adherence to the high-inflammatory DP and higher cIMT [41]. Interestingly, we observed an association between the high B-vitamin DP and CRP. This suggests that the effect of diet on CVD may be mediated by inflammation which in turn can promote endothelial dysfunction and atherogenesis. We also observed an association between higher adherence to the B-vitamin DP and lower triglyceride levels. In Whitehall II, greater adherence to a RRR DP defined by serum total and HDL-cholesterol, and triglycerides was found to predict coronary heart disease [51]. Similarly, another study found that young people with type 1 diabetes who adhered a RRR DP using triglycerides as one intermediate variable (other intermediate variables: LDL, systolic blood pressure, HbA1c, CRP and waist circumference) had higher arterial stiffness compared to those scoring lower on the DP [52].

RRR is one method to identify DPs. It has advantages over other DP methods such as PCA as it can examine specific mechanistic links between diet and outcomes of interest [18]. It is important to note that the DPs produced in our study are specific to the bespoke pathway under investigation i.e. via intakes of folate, B12 and Hcy plasma concentrations. To our knowledge, no other study has used a combination of plasma and dietary intake intermediate variables in RRR. Through this study we demonstrated the limitations of using weakly correlated intermediate variables.

In conclusion, although we found no consistent evidence for an association between a high B-vitamin DP and vascular function, we did observe an association with CRP and triglycerides in secondary analyses. Further analyses using well correlated intermediate variables are required to refine investigations into diet and CVD.

## Conflicts of interest

All authors have no potential conflict of interest.

## Author's contributions

JM and SR had full access to all of the data in the study and take responsibility for the integrity of the data and the accuracy of the data analysis. Study concept and design: SR, GA & JM. Acquisition of data: AW, RH. Analysis and interpretation of data: JM, GA, SR. Drafting of the manuscript: JM. Critical revision of the manuscript for important intellectual content: JM, GA, JG, AW, RH. Statistical analysis: JM. Administrative, technical or material support: JW, AW, RH. Study supervision: GA, SR. All authors read and approved the final manuscript.

## Disclaimers

All authors have nothing to declare.

## Sources of support

The British Medical Association (BMA/JL/2013/SR) and Medical Research Council (HNR/DR/2014/SR) funded this project via BMA Research Charity Foundations and the MRC Directors’ reserve Competition, but did not play a role in the design, collection, analysis or interpretation of the data, or writing of the report. The MRC functioned as the lead sponsor for the study. With the exception of standard reporting requirements for progress to the funders, all design, analyses, interpretation and the preparation of the manuscript was entirely independent of the funders and/or sponsors.

The NNEdPro Global Centre for Nutrition and health also provided in-kind support towards this work but did not in any way impact the scientific independence of the analyses or manuscript.
