# Supplementary material for: A dietary pattern derived using B-vitamins and its relationship with vascular markers over the life course
Source: Clin Nutr. 2019 Jun;38(3):1464–73. doi: 10.1016/j.clnu.2018.06.969 (PMC6546956; doi:10.1016/j.clnu.2018.06.969)
Supplement: Multimedia component [file mmc1.docx]

| **Supplementary Materials** | |
| --- | --- |
| **Supplementary table 1**. Food Groups | |
| **Food Group** |  |
| Alcohol | Beverages - Alcohol - Alcopops  Beverages - Alcohol - Beer  Beverages - Alcohol - Fortified wine  Beverages - Alcohol - Low alcohol beer  Beverages - Alcohol - Spirits & Liqueur |
| Wine | Beverages - Alcohol - Wine |
| Soft Drinks | Beverages - Carbonated soft drinks |
| Coffee & Tea | Beverages - Coffee  Beverages - Powdered Beverages (cocoa, Horlicks, Bonvita, Ovaltine, etc)  Beverages - Tea  Beverages - Water (still, tap, sparkling, flavoured) |
| Fruit based drinks | Beverages - Fruit based drinks  Beverages - Fruit based drinks - Fruit juice drinks  Beverages - Fruit based drinks - Pure fruit juice & smoothies |
| Squashes and fruit concentrates | Beverages - Fruit based drinks - Squashes & fruit concentrates |
| White bread | Breads - Other bread  Breads - White |
| Brown & wholemeal bread | Breads - Crisp Breads, e.g. Rivetas, Grissini, Toast Melba  Breads - Wholemeal  Breads - Brown/Granary/Wheatgerm |
| High fibre breakfast cereal | Breakfast cereals - Oat based cereals  Breakfast cereals - Other breakfast cereals - high fibre (equal or >3g/40g portion) |
| Low fibre breakfast cereal | Breakfast cereals - Other breakfast cereals - low fibre |
| Other cereal and cereal dishes | Cereals & cereal dishes - Other cereals & dishes |
| Pasta & pasta dishes | Cereals & cereal dishes - Pasta & pasta dishes |
| Pizza | Cereals & cereal dishes - Pizza |
| Rice & rice dishes | Cereals & cereal dishes - Rice & rice dishes |
| Chocolate confectionary | Confectionary - Chocolate based products |
| Sugar confectionary | Confectionary - Sorbets & lollies  Confectionary - Sugar based products |
| Cheese | Dairy products - Cheese, incl. cottage cheese  Dairy products - Cream & fromage frais |
| Ice cream | Dairy products - Ice cream & dairy desserts  Dairy products - Ice cream & dairy desserts - full fat products  Dairy products - Ice cream & dairy desserts - reduced fat products |
| Full Fat Yoghurt | Dairy products - Yoghurt & drinking yoghurts, incl. buttermilk and probiotics - full fat products |
| Low-fat Yoghurt | Dairy products - Yoghurt & drinking yoghurts, incl. buttermilk and probiotics - reduced or low fat products |
| Egg & egg dishes | Egg & egg dishes |
| Animal based fats | Fats - Animal based fats (solid)  Fats - Butter |
| Oils | Fats - Oils |
| Fats - Plant based fats | Fats - Plant based fats (solid) - Full fat  Fats - Plant based fats (solid) - Low fat  Fats - Plant based fats (solid) - Reduced fat |
| Fish & fish dishes | Fish & fish dishes - Oily fish  Fish & fish dishes - Shellfish  Fish & fish dishes - White fish, incl. tuna |
| Fruit | Fruit - Canned & cooked  Fruit - Dried  Fruit - Fresh |
| Red meat | Meat - red - Beef & veal & dishes  Meat - red - Lamb & dishes  Meat - red - Other red meat, e.g. rabbit, venison  Meat - red - Pork & dishes |
| White meat | Meat - white - Chicken & turkey & dishes  Meat - white - Other game birds, (e.g. duck, goose, pheasant) & dishes |
| Low fat Milk & plant based milk | Milk - 1% milk  Milk - Other - plant based, e.g. rice, soy  Milk - Semi-skimmed milk  Milk - Skimmed milk |
| Whole Milk & animal based | Milk - Milk based drinks, e.g. flavoured milks  Milk - Whole milk |
| Herbs, salt & artificial sweeteners | Miscellaneous - Artificial sweeteners  Miscellaneous - Dried herbs & spices & pastes  Miscellaneous - Salt and salt substitutes |
| Nutrition Powders & drinks | Nutrition Powders & drinks |
| Nuts & Seeds | Nuts & Seeds (incl. peanut butter) |
| Offal | Offal - Liver & dishes  Offal - Other offal & dishes, e.g. Haggis, faggots |
| Potatoes | Potatoes - Potato products - other  Potatoes - Potatoes |
| Chutney & Pickles | Preserves - Chutney & Pickles (incl. gherkins, pickled onions etc) |
| Sugar and preserves | Preserves - Jam & Marmalade  Sugars - Pure sugars |
| Processed Meat | Processed meat - Bacon & ham  Processed meat - Other processed meats  Processed meat - Processed pies  Sausages & burgers & kebab |
| Pulses Lentils | Pulses/Lentils - Baked beans  Pulses/Lentils - Pulses/lentils |
| Sauces & accompaniment | Sauces & accompaniment - Cooking sauces, incl. gravies, pesto, cooking sauces for pasta and rice dishes  Sauces & accompaniment - Dressings & Mayonnaise  Sauces & accompaniment - Other sauces, incl. brown sauce, soy sauce, ketchup, mint sauce, vinegar |
| Savoury Snacks | Savoury Snacks - Cereal based snacks  Savoury Snacks - Potato based snacks  Savoury Snacks - Savoury biscuits & crackers  Savoury Snacks - Vegetable based snacks |
| Soups | Soups - Canned & fresh & homemade  Soups - Dried |
| Honey | Sugars - Other, incl. syrups, honey |
| Sweet cereal products | Sweet cereal products - Biscuits  Sweet cereal products - Cereal based puddings (not milk)  Sweet cereal products - Milk based puddings  Sweet cereal products - Pastries, Buns & Pies |
| Cereal bars | Sweet cereal products - Cereal bars |
| Vegetables | Vegetables - Brassicacea  Vegetables - Other  Vegetables - Tomatoes  Vegetables - Tomatoes - Puree & sun-dried  Vegetables - Tomatoes - Raw & canned  Vegetables - Yellow & red & dark green leafy vegetables |

| **Supplementary Table 2**. Comparison eligible versus ineligible participants for current study (of the 2,229 responding to the 60-64 year follow up) | | | |
| --- | --- | --- | --- |
|  | Ineligible participants  (*n*=667) | Eligible participants  (*n*=1,562) | *P*_difference_^†^ |
| % Female | 51.42 | 52.43 | 0.66 |
| PWV (m/s) at 60-64 years, mean (SD) | 8.52 (1.4) | 8.18 (1.52) | 0.01 |
| cIMT (mm) at 60-64 years, mean (SD) | 0.70 (0.11) | 0.69 (0.13) | 0.01 |
| % High SEP (I & II) at 53 years (or 43 years if missing) | 32.32 | 53.58 | <0.001 |
| BMI at 60-64 years, kg/m^2^, mean (SD) | 28.85 (5.51) | 27.54 (4.55) | <0.001 |
| % Current smoker at 60-64y | 19.94 | 10.80 | <0.001 |
| % Physically inactive since 1982 to 2006-2009 | 24.89 | 12.75 | <0.001 |
| % Receiving anti-hypertensive medication at 60-64 years | 35.60 | 27.64 | <0.001 |
| % Receiving lipid-lowering medication at 60-64 years | 28.79 | 20.87 | <0.001 |
| % Diabetes at 60-64 years | 20.51 | 25.61 | 0.03 |
| Systolic blood pressure (mmHg) at 60-64 years, mean (SD) | 138.08 (18.41) | 135.61 (18.16) | <0.001 |
| Diastolic blood pressure (mmHg) at 60-64 years, mean (SD) | 79.22 (10.12) | 77.21 (9.82 | <0.001 |
| Total cholesterol (mmol/L) at 60-64 years , mean (SD) | 5.60 (1.21) | 5.69 (1.19) | 0.002 |
| LDL cholesterol (mmol/L) at 60-64 years, mean (SD) | 3.40 (1.04) | 3.53 (1.02) | <0.001 |
| HDL cholesterol (mmol/L) at 60-64 years, mean (SD) | 1.56 (0.43) | 1.60 (0.40) | 0.001 |
| Triglycerides (mmol/L) at 60-64 years , geometric mean (95% CI) | 1.29 (1.24, 1.35) | 1.12 (1.09, 1.14) | <0.001 |
| C-reactive protein (mg/L) at 60-64 years, geometric mean (95% CI) | 2.71 (2.51, 2.92) | 2.18 (2.08, 2.28) | <0.001 |
| ^†^ χ^2^ test, ttest or Wilcoxon rank sum as appropriate | | | |

| **Supplementary table 3**. Correlations between intermediate variables and dietary patterns | | | |
| --- | --- | --- | --- |
|  | **DP1** | **DP2** | **DP3** |
| **% Explained variation*** | |  |  |
| folate ug/kcal | 12.37 | 12.39 | 12.60 |
| B12 ug/kcal | 1.00 | 2.53 | 4.19 |
| Hcy | 0.13 | 3.69 | 4.47 |
| Total | 4.50 | 6.20 | 7.09 |
| **Dependent variable weights** | |  |  |
| folate ug/kcal | 0.96 | 0.07 | -0.28 |
| B12 ug/kcal | 0.27 | -0.55 | 0.79 |
| Hcy | 0.10 | 0.83 | 0.54 |
| *Variation in intermediate variables explained by the dietary pattern, cumulative values  DP, dietary pattern | | | |

| **Supplementary table 4**. Distribution of folate, vitamin B12 and energy per year, mean (SD) | | | | |
| --- | --- | --- | --- | --- |
|  | **n** | **Folate ug/d** | **Vitamin B12 ug/d** | **Energy kcal/d** |
| **36 years** | 2,411 | 218.1 (94.1) | 6.82 (7.67) | 2037.2 (655.6) |
| **43 years** | 2,256 | 278.1 (94.8) | 6.35 (5.21) | 2139.0 (625.2) |
| **53 years** | 1,772 | 294.7 (97.7) | 6.33 (4.46) | 1982.1 (510.0) |
| **60-64 years** | 1,869 | 348.8 (212.6) | 8.97 (30.44) | 1879.3 (458.6) |

**
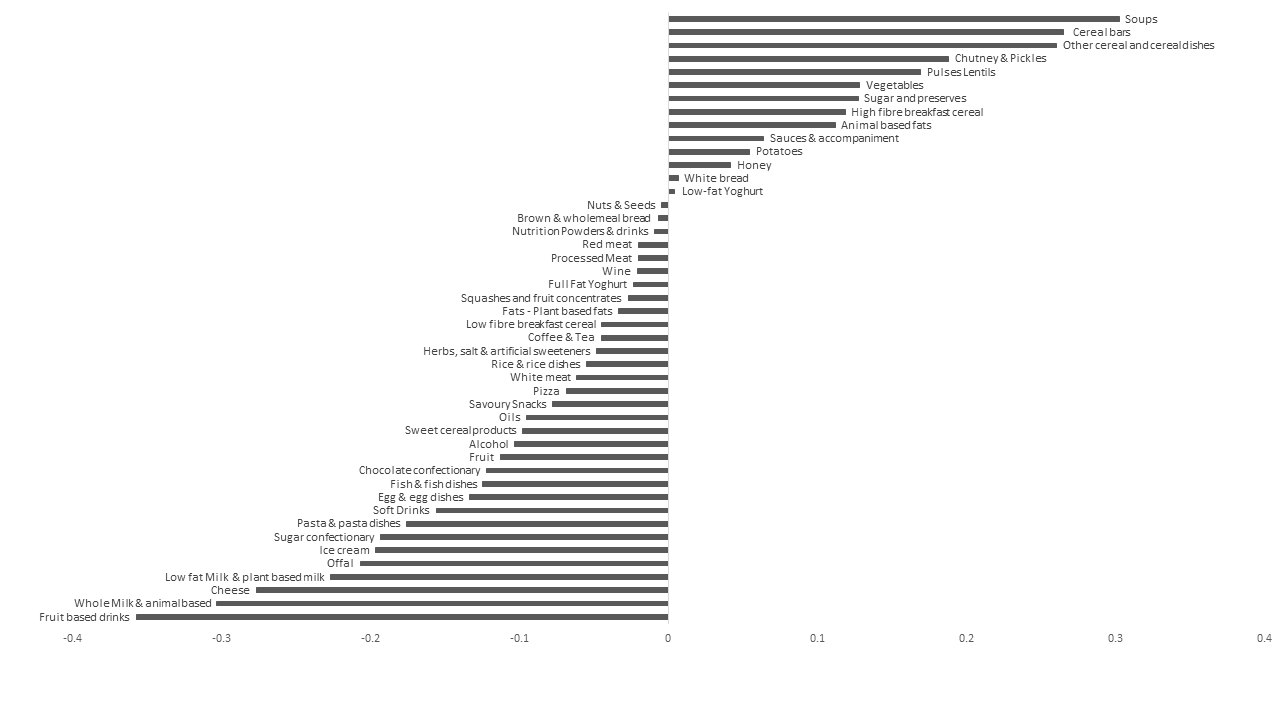
**

**Supplementary figure 1.** Factor loadings for a high homocysteine and high folate dietary pattern (DP 2) at 60-64 years

**
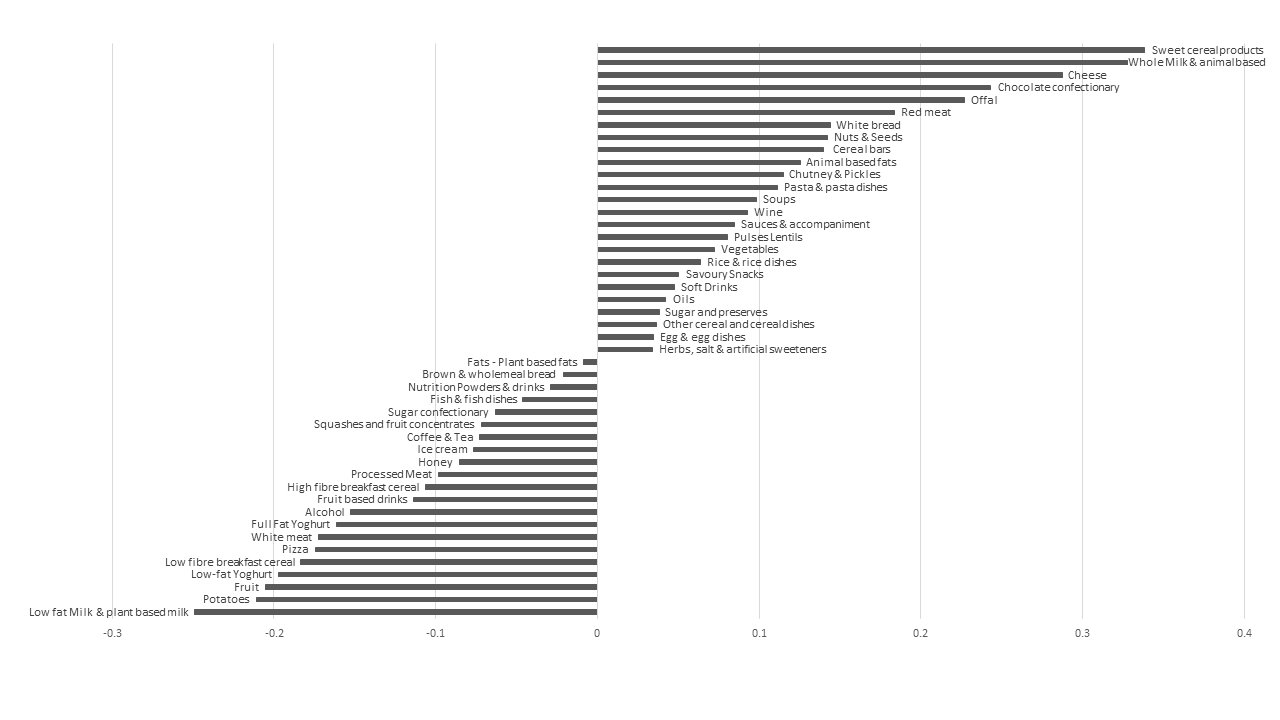
**

**Supplementary figure 2.** Factor loadings for high homocysteine and high B12 dietary pattern (DP 3) at 60-64 years
